# Comparison of Dermatoglyphic Pattern among Cleft and Noncleft Children: A Cross-sectional Study

**DOI:** 10.5005/jp-journals-10005-1444

**Published:** 2017-02-27

**Authors:** Sandeep S Mayall, Seema Chaudhary, Harsimran Kaur, Naveen Manuja, Telegi Ravishankar, Ashish A Sinha

**Affiliations:** 1 Senior Lecturer, Department of Pedodontics and Preventive Dentistry, Teerthanker Mahaveer Dental College and Research Centre, Moradabad Uttar Pradesh, India; 2 Professor and Head, Department of Pedodontics and Preventive Dentistry, Kothiwal Dental College and Research Centre, Moradabad, Uttar Pradesh, India; 3 Reader, Department of Pedodontics and Preventive Dentistry, Kothiwal Dental College and Research Centre, Moradabad, Uttar Pradesh, India; 4 Professor, Department of Pedodontics and Preventive Dentistry, Kothiwal Dental College and Research Centre, Moradabad, Uttar Pradesh, India; 5 Reader, Department of Pedodontics and Preventive Dentistry, Kothiwal Dental College and Research Centre, Moradabad, Uttar Pradesh, India; 6 Reader, Department of Pedodontics and Preventive Dentistry, Kothiwal Dental College and Research Centre, Moradabad, Uttar Pradesh, India

**Keywords:** Dermal appendages, Dermatoglyphics, Epidermal ridges, Orofacial cleft

## Abstract

**Background:**

Oral clefts are among the common congenital birth defects with a broad phenotypic gamut. Since the epidermal ridges of the fingers and palms as well as the facial structures like lip, alveolus, and palate are formed from the same embryonic tissues during the same embryonic period, the genetic and environmental factors responsible for causing cleft lip and palate might also affect dermatoglyphic patterns.

**Aim:**

Thus, study was undertaken to compare the dermato-glyphic pattern of children with orofacial clefts and normal children and to determine the correlation of dermatoglyphics with orofacial clefts.

**Materials and methods:**

Total study sample consisted of 120 children in the age group of 3 to 16 years being divided into study and control groups. Dermatoglyphic data obtained from both control and study groups were then subjected to statistical analysis.

**Results:**

Statistically no significant difference was found in the dermatoglyphic pattern and atd angle for both the groups.

**Conclusion:**

It was observed that dermatoglyphics in orofacial clefts may not be distinctive. Further, large-scale studies are recommended to confirm the same.

**How to cite this article:**

Mayall SS, Chaudhary S, Kaur H, Manuja N, Ravishankar T, Sinha AA. Comparison of Derma-toglyphic Pattern among Cleft and Noncleft Children: A Cross-sectional Study. Int J Clin Pediatr Dent 2017;10(3):245-249.

## INTRODUCTION

Craniofacial anomalies, in particular cleft lip and palate, are major human birth defects with a worldwide frequency of 1 in 700. Cleft lip is an abnormality in which the lips are not completely formed, whereas cleft palate occurs when the roof of the palate is not fused, leaving a communication that may or may not extend into the nasal cavity.^1^ Dermatoglyphics, introduced in 1926 by Dr Harold Cummins—the father of fingerprint analysis, is applied to the study of the naturally occurring patterns of the surface of the hands and feet.^2^ Dermal ridge differentiation takes place early in fetal development. The resulting ridge configurations are genetically determined and are influenced by environmental factors.^3^ The development of the primary palate and the lip is completed by the 7th week of intrauterine (i.u.) life and that of secondary palate by 12th week of i.u. life. The dermal ridges, developing in relation to the alveolar pads, are formed by the 6th week of gestation and reach maximum size between 12th and 13th weeks. This means that the genetic message contained in the genome—normal or abnormal—deciphered during this period might also be reflected by dermatoglyphics.^4^ Hence, this study was undertaken to observe the differences in the dermato-glyphic pattern between the children with orofacial clefts and normal children as well as to determine the relevance of dermatoglyphics in studying the genetic etiology of orofacial clefts.

## MATERIALS AND METHODS

The study was conducted in the Department of Pedodon-tics and Preventive Dentistry, Kothiwal Dental College, Moradabad in collaboration with the Department of Oral and Maxillofacial Surgery, Kothiwal Dental College, Moradabad, Uttar Pradesh, India.

### Sample Selection

Data were collected from 120 subjects in the age group of 3 to 16 years with no gender consideration.

### Inclusion Criteria

- *Age:* 3 to 16 years
- *Group I: Control group:* 60; Normal healthy children without any congenital or medical anomalies
- *Group II: Study group:* 60; nonsyndromic children with orofacial clefts

**Fig. 1:**
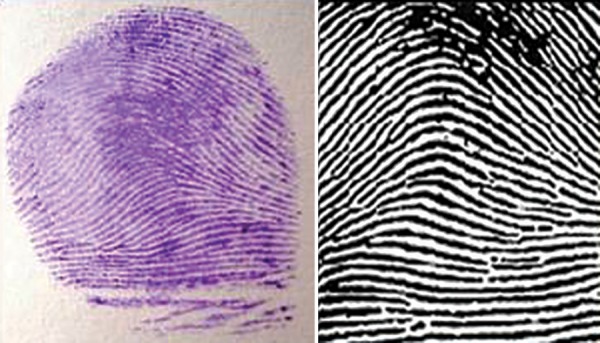
Arch Pattern—impression and diagrammatic Representation

**Fig. 2:**
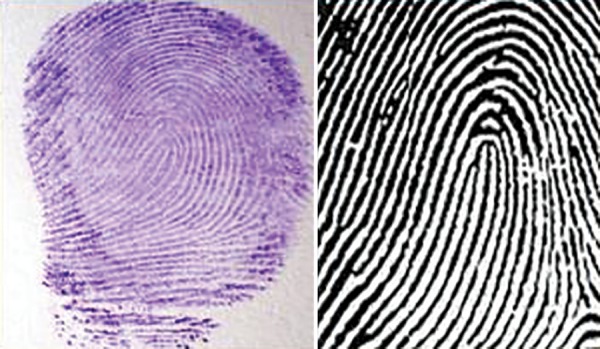
Loop pattern—impression and diagrammatic representation

**Fig. 3:**
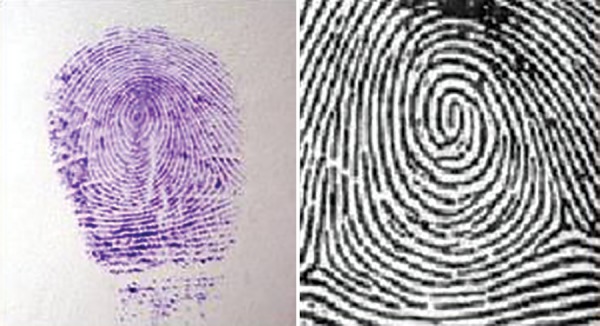
Whorl pattern—impression and diagrammatic representation

**Fig. 4:**
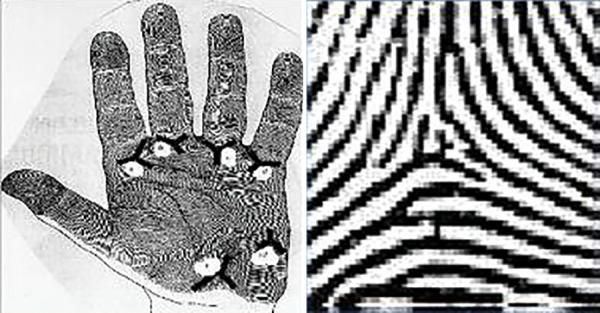
Triradii locations (a, b, c, d, t) and tri radius

**Fig. 5:**
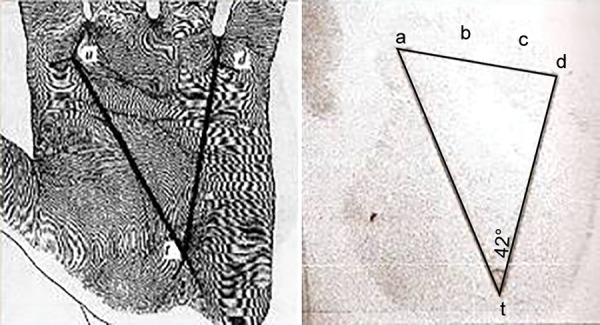
Angle ’atd’ and determination of atd angle

### Data Collection

Fingerprints and palm prints were individually taken from each subject using the ink method with the black duplicating ink manufactured by Kores Limited Golden Stamp Pad, Ashoka Company Marketing Co, Ramesh Nagar, New Delhi and were analyzed by using a hand-magnifying glass.

Fingerprint Pattern Analysis

In the present study, 1,200 digital prints were obtained from the bilateral fingers of all 120 subjects, 60 from study group and 60 from the control group and were analyzed with the help of hand-magnifying glass. Based on the ridge configuration, three basic types of ridge patterns were encountered:

1. Arch pattern (Fig. 1)
2. Loop pattern (Fig. 2)
3. Whorl pattern (Fig. 3)

Palm Print Analyses (atd Angle)

The triradius (Fig. 4) is the meeting of the ridges following in three directions, where ridges from angles of approximately 120° with one another. The atd angle is formed by a line drawn from the digital triradii "a" to the axial triradii "t" and from this to the digital triradii "d" (Fig. 5). In this way, 240 atd angles were obtained from all the subjects and classified into three groups: <45°, 45 to 56°, and >56°.

The finger and palm areas were analyzed for derma-toglyphic pattern analysis and "atd" angle. Interpretation of patterns was carried out according to Cummins and Mildo^5^ and Penrose.^6^

### Statistical Analysis

The collected data were interpreted and subjected to statistical analysis using Statistical Package for the Social Sciences, version 15.0, a statistical analysis software. The values were represented in number (%) and mean ± standard deviation (SD).

**Table 1:** **Table 1:** Comparison of mean number of different dermatoglyphic patterns in two groups

		*Group I* *(n = 60) (control)*				*Group II* *(n = 60) (study)*				*Statistical* *significance*			
*Pattern*		*Mean*		*±SD*		*Mean*		*± SD*		*t-value*		*p-value*	
Arch		0.58		1.33		0.53		0.89		0.242		0.809	
Loop		5.52		2.40		6.23		2.63		1.559		0.122	
Whorl		3.67		2.74		3.22		2.72		0.903		0.450	

**Table 2:** **Table 2:** Comparison of two groups according to atd angle

		*Group I (n = 60)*				*Group II (n = 60)*			
*L* *atd (°)*		*No.*		*%*		*No.*		*%*	
*Left hand*									
<45°		43		72.9		39		65.0	
45°-56°		16		27.1		17		28.3	
>56°		0		0		4		6.7	
		χ^2^ = 4.217 (df = 2); p = 0.121							
*Right hand*									
<45°		48		80.0		41		68.3	
45°-56°		11		18.3		18		30.0	
>56°		1		1.7		1		1.7	
		χ^2^ = 4.217 (df = 2); p = 0.121							

**Graph 1:**
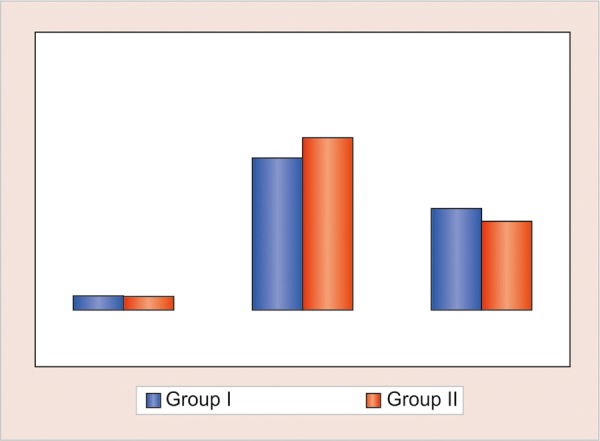
Comparison of mean number of different dermatoglyphic patterns in two groups

## RESULTS

**Graph 2:**
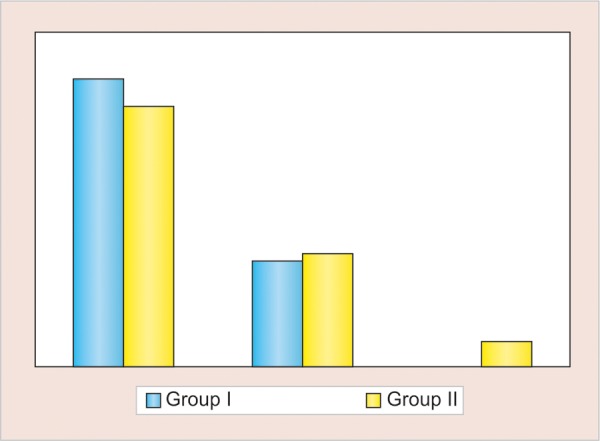
Left side atd angle

**Graph 3:**
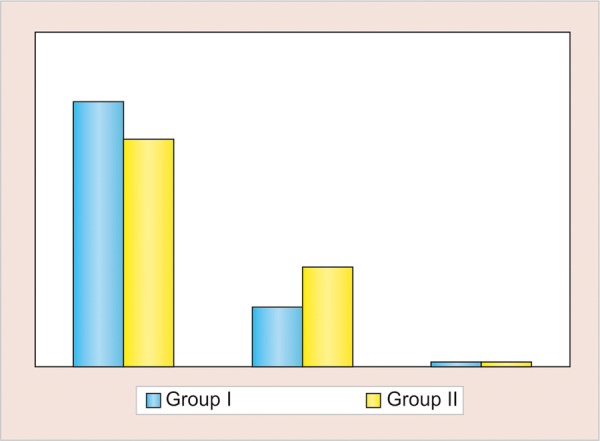
Right side atd angle

Comparison of mean number of different dermatoglyphic patterns in two groups revealed that the mean number of arches and whorls were found to be higher in group I (control), while mean number of loops were found to be higher in group II (study) (Table 1 and Graph 1). No statistically significant intergroup difference was seen for any of the three patterns (p > 0.05). Both for the left and right hand of group I (control), the atd angle was <45° in 72.9% and 80% of the subjects respectively, followed by an atd angle of 45 to 56° and least percentage was for atd angle of >56°. Both for the left and right hand of group II (study), the atd angle of <45° was 65 and 68.3% respectively (Table 2, Graphs 2 and 3). No statistical significant difference was found in the atd angle among the two groups.

## DISCUSSION

Since ages, features of hands have fascinated innumerable theologians, doctors, and laymen, but it has been recently known that dermatoglyphics can act as a window of congenital anomalies.^3^ According to Yamagata, any deviation in the dermatoglyphics features indicates a genetic difference.^7^ Dermatoglyphics, in recent times, have proven to be instrumental in identifying specific congenital syndromes of orofacial region, like cleft lip and palate. In human, the embryogenesis of dermal appendages and oral cavity occurs almost during the same time. The development of the primary palate and lip is completed by the 7th week of IU life and that of secondary palate 12th week. The dermal ridges develop in relation to alveolar pads, which are formed by the 6th week of gestation and reach maximum size between 12th and 13th weeks. Abnormalities in the epidermal ridges may result from genetic alterations occurring around the first trimester. This means that the genetic message contained in the genome-normal or abnormal, deciphered during this period, is reflected by dermatoglyphics.^8^

Cleft lip with or without cleft palate is a congenital anomaly with a prevalence that varies by population 1:500 to 1:2000.^9^ Over many decades, the etiology and mode of transmission of congenital cleft lip and palate anomalies has been instigative.^8^ While most of the cases of this malformation have a polygenic mode of inheritance, a certain proportion results from rare mutant gene and chromosomal aberrations and unknown exogenous factors.^10,11^ However, the exact etiology and mechanism of transmission of these malformations are still ambiguous.^2^

The epidermal ridges of the fingers and palms as well as the facial structures like the lip, alveolus, and palate are formed from the same embryonic tissues during the same embryonic period (6-9 weeks). Kanematsu et al^12^ stated that genetic and environmental factors that are responsible for causing cleft lip and palate may also cause peculiarities in the dermatoglyphic patterns.

Despite the high prevalence of cleft lip and palate reports, studies regarding the relationship between cleft lip with or without cleft palate and dermatoglyphics deviations are relatively sparse.^13^ Thus, this study was instigated to evaluate any differences in the dermato-glyphic pattern among cleft and noncleft children.

The study group consisted of nonsyndromic children with orofacial clefts and control of normal healthy children without any medical or congenital anomalies because syndromes and other anomalies may alter the dermatoglyphic pattern.^2^ Hands of the subjects were thoroughly washed and dried before taking prints. This was done to remove the dirt from the hands. Dermatoglyphic data were collected using the ink method.^2^ Rolled and repeated prints were taken to avoid incomplete configuration and erroneous classification.^13^

Soon after the print was taken, it was examined with a hand-magnifying lens for details and clarity in the different fingers and palm areas. The finger and palm areas were analyzed for dermatoglyphic pattern analysis and "atd" angle. Interpretations of patterns were carried out according to Schaumann and Alter,^3^ Cummins and Mildo,^5^ and Penrose.^6^

### Fingerprint Analysis (Dermatoglyphic Pattern Analysis)

On comparison of mean number of different dermato-glyphic pattern in two groups, the mean number of arches and whorl was found to be higher in group I (control) as compared with group II (study); while number of loops was found to be higher in group II as compared with group I. No statistically significant intergroup difference was found for any of the three patterns. These results were synonymous to the results by Silver^14^ and Neiswanger et al^15^ in which pattern frequencies and "atd" angle did not differ statistically between cleft and normal children. Contrary to the results of our study, Yamagata^16^ and Balgir^8^ had a lower frequency of whorl patterns and a higher frequency of ulnar loops in the fingers of children with orofacial clefts. Mathew et al^2^ observed that the oral cleft children had a significantly higher number of ulnar loops as compared with the normal children with higher frequency of whorls.

In the mentioned study, there was no significant difference found between any of the dermatoglyphic configurations of the orofacial cleft group and controls. It seems that orofacial cleft is a congenital anomaly whose development basis seems to be independent of a production of aberrant dermatoglyphic patterns. These findings signify that although genetic factors play a vital role in determining ridge configurations, nongenetic factors also exert major influence in distinguishing the same.^17^

### Palm Print Analysis (atd Angle)

With the help of hand-magnifying lens, "a," "t," and "d" triradii were located and atd angle was determined by a line drawn from the digital triradii "a" to the axial triradii "t" and from this to the digital triradii "d." A triradii is formed by the confluence of three ridge system; the geometric center of each triradii is termed as triradial point. In this way, 240 atd angles values were obtained from all the subjects and atd angle was measured and classified into three groups: <45°, 45 to 56°, and <56°.

In the present study, values observed for atd angle for orofacial cleft and normal children for both the hands were in the range of <45°, followed by 45 to 56° and then 56° (45° > 45°-56° > 56°). The difference was not statistically significant between the study group and the control group. Similar results were reported by Balgir^8^ and Neiswanger et al,^15^ where the difference in atd angle between cleft lip with or without palate and cleft palate patients was not statistically significant. Contrary to our study, Mathew et al^2^ observed an atd angle of >45° in orofacial cleft children, while <45° in normal children.

Dermatoglyphics data may prove to be of biomedical significance in certain congenital anomalies; the impact of environmental factors on distinguishing the same cannot be ignored.

## CONCLUSION

From the aforementioned study, it can be suggested that dermatoglyphics in orofacial cleft children are not distinctive. Nevertheless, further extrapolations are recommended to confirm the same.
